# Single-cell RNA sequencing reveals early cell dynamics of MSC-based therapy in long bone critical-size defects in mice

**DOI:** 10.1016/j.jot.2025.08.007

**Published:** 2025-09-09

**Authors:** Ning Zhang, Jie Yuan, Xueping Li, Ni Su, Yiyun Wang, Shuxian Chen, Ejun Huang, Qi Gao, Fan Yang, Simon Kwoon-Ho Chow, Stuart B. Goodman

**Affiliations:** a Musculoskeletal Research Laboratory, Department of Orthopaedics & Traumatology, The Chinese University of Hong Kong, China; b Department of Orthopaedic Surgery, Stanford University School of Medicine, California, USA; c Li Ka Shing Institute of Health Sciences, The Chinese University of Hong Kong, China; d International Cancer Center, Shenzhen University Medical School, Guangdong, China; e Department of Biomedical Engineering, University of Kentucky, Lexington, KY, USA; f Yangzhi Rehabilitation Hospital (Shanghai Sunshine Rehabilitation Center), School of Medicine, Tongji University, Shanghai, China; g Department of Bioengineering, Stanford University, California, USA

**Keywords:** Single-cell RNA sequencing, Mesenchymal stem cell, Bone defect, Bone healing

## Abstract

**Background:**

Bone defects resulting from various causes present significant challenges in obtaining robust bone healing. This clinical scenario is particularly difficult in cases involving large bone defects, often leading to delayed union or non-union. Autogenous bone graft is the gold standard, but it is limited by the quantity and quality of available bone. Mesenchymal stem cells (MSCs) have shown promise in enhancing bone defect healing; however, the mechanisms by which MSCs modify the local bone microenvironment and interact with other cells early in the healing process are not fully understood. Elucidating and modulating the early biological events relevant to the healing of bone defects could lead to novel therapies to obtain a more expeditious and complete outcome.

**Methods:**

Critical-size femoral defects were created in 10 to 12-week-old BALB/c male mice and fixed with an external fixation device. Four weeks after the generation of the defect, secondary surgeries were performed. Mice were randomized into three groups based on the secondary surgery: Empty group - surgery was performed without implanting scaffolds or cells. Sc group - a 2 mm diameter cylindrical microribbon (μRB) scaffold was implanted into the defect site. Sc + MSC group - a scaffold embedded with MSCs was implanted into the bone defect site. One week after the secondary surgeries, the entire tissue within the bone defect site was harvested for single-cell RNA sequencing (scRNA-seq).

**Results:**

Uniform manifold approximation and projection (UMAP) plots with quality filtered cells from three groups were used to identify the cell distributions in the defects. We identified thirteen populations and annotated each cluster using UMAP with Louvain clustering on combined single cells of three groups based on marker gene expression. Different cell compositions were revealed, especially the proportion of various types of immune cells in the Sc vs Sc + MSC groups. MSCs and osteoblastic lineage cells (MSC/Osteo), and osteoclasts were almost exclusively found in the Sc + MSC group. Differential gene expression and pathway analysis in major cell populations identified immune cell changes and inflammatory changes in the presence of implanted MSCs. Cell–cell communications revealed a greater number of interactions between different cell types in the Sc and Sc + MSC groups. More interactions among MSCs, macrophages, and T cells were observed in Sc + MSC groups. MSC demonstrated the highest outgoing interaction strength in all groups.

**Conclusions:**

In the critical-size bone defect model, a combination of MSCs with μRB scaffolds showed an increased presence of mesenchymal lineage cells and promoted the further recruitment of macrophages and osteoclasts at 1 week. This alteration in the local immune landscape and microenvironment could enhance the cellular dynamics of critical cell populations that are important to osteogenesis. Optimizing this cellular crosstalk early in the healing process could potentially augment MSC-based therapies for subsequent bone regeneration of critical-size bone defects.

**The translational potential of this article:**

The results of our study provide a detailed transcriptional roadmap for local immune modulation by MSCs in scaffolds, supporting the optimization of robust strategies for MSC-based treatments of long bone critical-size defects in future clinical applications.

## Graphical abstract

**Figure:**
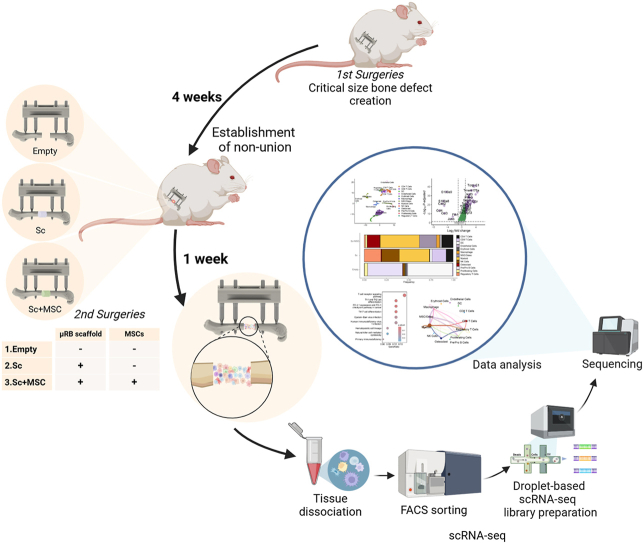

## 1 Introduction

Bone defects resulting from trauma, complex fractures, infections, tumors, and periprosthetic osteolysis present significant challenges. Healing these defects is particularly difficult in cases involving advanced age and large defect sizes, often leading to delayed union or non-union [1]. The direct cost of treating a non-union is estimated at $45,000 USD, with a total annual cost of $9.2 billion in the US [2]. Globally, direct treatment costs an average of approximately US$11,333 per case [2]. Treating critical-size or large bone defects remains a significant clinical challenge for surgeons. Autogenous bone grafting is the most common method for bone defect treatment in clinical practice, with over 2 million bone graft procedures performed annually worldwide [3]. Despite over a century of successful clinical use, autologous bone transplantation is often limited by the quantity and quality of available bone [4]. Additionally, harvesting autologous bones can lead to complications such as infection, localized pain, and long-term disability. Healing critical-size defects in the lower extremities with large, segmental, vascularized bone transfers can take 12–18 months and carries a high risk of refracture [5]. Due to these limitations, new strategies need to be developed through bone tissue engineering technology.

Mesenchymal stem cells (MSCs) from various tissues have shown promise in enhancing bone defect healing [[6], [7], [8]]. This is achieved through various methods, including pharmacology-based rescue and mobilization of endogenous MSCs, systemic or local infusion of MSCs for cytotherapy, biomaterials-based approaches, and others [7]. Pre-implantation processes may be used to guide MSC differentiation and tissue formation more effectively during MSC-based therapies for long bone defects [9]. Some of these strategies to enhance the function of harvested MSCs include preconditioning the cells, exposing the cells to low-oxygen environments, and genetic manipulation of the cells. The use of MSCs or genetically modified MSCs for the treatment of critical-sized bone defects has shown encouraging results [[10], [11], [12], [13], [14], [15]]. However, due to FDA regulations on "minimal manipulation" criteria for human cells, tissues, and cellular and tissue-based products (HCT/Ps), these treatments face significant scientific, regulatory, and financial hurdles before they can be widely used [9].

There is limited knowledge of the mechanisms by which transplanted MSCs modify the local bone microenvironment and how these cells impact the essential intercellular crosstalk to enhance bone healing. Previously, we investigated the dynamics of bone graft transplantation and mesenchymal stem cell therapy during bone defect healing in a murine critical-sized femur defect using mass cytometry by time of flight (CyTOF) [16]. We observed active recruitment of endogenous MSCs to the defect site and the diversity of immune cells that populated the defect. Changes in this niche microenvironment after MSC implantation are important for facilitating tissue repair. Interactions between MSCs and local cells are essential for activating or licensing MSCs and providing biological cues to guide function. Understanding these mechanisms is crucial for optimizing MSC-based therapies for long bone critical-sized defects.

In this study, we established a model of a long bone critical-size defect and implanted MSCs within a gelatin microribbon (μRB) scaffold 4 weeks after the creation of the bone defect. The μRB scaffold has previously been shown to accelerate stem cell-based bone regeneration in a mouse critical-size cranial defect model [17]. We employed 10X Genomics scRNA-seq to examine cell heterogeneity and the transcriptional roadmap, aiming to map the early differential cellular microenvironment of the MSC-based therapy.

## 2 Materials and methods

### 2.1 Animal surgery

10–12 weeks old BALB/c male mice were purchased from Jackson Laboratory. Stanford University's Administrative Panel on Laboratory Animal Care approved this surgical protocol (Protocol number: APLAC-26950). The right femur was stabilized with a custom murine external fixator and 2 mm critical-size femoral defects were made using a special jig (RISystem AG, Switzerland). The mice were randomized into three groups: empty defect (Empty) group (n = 5), Scaffold without MSC (Sc) group (n = 5), and Scaffold seeded with MSCs (Sc + MSC) group (n = 5). Four weeks after the generation of the defect, secondary surgeries were performed. For the Empty group, the sham surgery is performed without implanting the scaffold. For the Sc group, a 2 mm diameter cylindrical μRB scaffold without MSCs was implanted into the defect site. For the Sc + MSC group, a μRB scaffold embedded with MSCs (details shown in the following parts) was implanted into the bone defect site. One week after the secondary surgeries, the entire tissue within the bone defect site was harvested for scRNA-seq. The healing of bone defect of the Empty group and Sc + MSC group (n = 5 each group) was also evaluated six weeks after the implantation (Fig. S1)

### 2.2 MSC isolation and culture

Bone marrow-derived MSCs were isolated according to a previously published method [[11], [12], [13], [14]]. Bone marrow was collected from the femurs and tibias of 10-week-old BALB/c male mice, then carefully suspended and passed through a 70-μm strainer, spun down and cultured in alpha-minimal essential medium (α-MEM, Thermo Fisher Scientific, Waltham, MA) supplied with 10 % MSC certified with fetal bovine serum (Invitrogen, Carlsbad, CA) and antibiotic antimycotic solution (100units of penicillin, 100 μg of streptomycin and 0.25 μg of Amphotericin B/ml; Hyclone, Thermo Fisher Scientific). The medium was replaced the next day to remove unattached cells, and the remaining cells were defined as passage 1. The immunophenotype of isolated MSCs (CD105+/CD73+/CD90.2+/Scal+/CD45−/CD34-/CD11b−) as defined by the International Society for Cell Therapy was characterized by LSR II flow cytometer (BD Biosciences, San Jose, CA) at passage 4. Cells between the 4 and 8 passages were used.

### 2.3 Gelatin μRB-based scaffold

The gelatin micro-ribbon scaffolds (μRBs) were fabricated through a wet spinning process according to previous reports [[11], [12], [13],^17^]. To fabricate scaffolds and embed MSCs within them, the μRBs were rehydrated in PBS containing 0.05 % LAP photo-initiator. After 1 h of incubation at 37 °C, trypsinized MSCs suspended in PBS were gently mixed with the μRBs at a concentration of 10 million cells/ml. The cell-laden μRBs were then filled into a 2 mm diameter × 2 mm height cylindrical mold and exposed to ultraviolet light (365 nm, 2 mW/cm^2^) for 4 min to produce macroporous scaffolds. After fabrication, the scaffolds were gently pushed out from the mold and kept in PBS for further applications. We assessed cell viability using a Live/Dead assay after one day of in vitro culture of the μRB scaffolds with MSCs, finding a viability of 88.96 % ± 7.77 % (Fig. S1D). The freshly made scaffolds were used for implantation into the defect sites as previously described [16].

### 2.4 Tissue processing and single-cell library preparation

Single-cell suspensions from each group were prepared. Briefly, tissues from the defect site (n = 5 for each group) were pooled, minced, and dissociated for 30 min at 37 °C with shaking at 210 rpm. Following incubation, the cell suspension was filtered through a 70 μm cell strainer into a clean 50 ml tube containing at least 20 ml of cold cell-suspension buffer. The suspension was then centrifuged at 200 g for 5 min at 4 °C. The supernatant was removed, and the cell pellet was resuspended in 1 ml of cell buffer. To the pellet, 10 ml of ACK lysis buffer was added and incubated at room temperature for 3 min. The cell suspension was centrifuged again at 200 g for 5 min at 4 °C. The supernatant was discarded, and the cells were resuspended in the FACS buffer. Samples were then analyzed by flow cytometry to sort and collect live cells based on the gating strategy (Fig. S2A). Briefly, single-cell suspensions were first gated to exclude debris and doublets using FSC-A vs. SSC-A and FSC-A vs. FSC-H plots. Singlet events were further gated using SSC-H vs. SSC-W to remove aggregates. Cells were then stained with Propidium Iodide (PI, 5 μl each per 100 μl of cells for 10 min at room temperature) to identify dead cells and PE-conjugated antibody to label erythroid lineage cells. Live, non-erythroid cells (PI^−^, PE^−^) were sorted and used for downstream single-cell library preparation. Using the Chromium instrument and the single-cell 3′ Reagent kit (V3.1), we prepared 10X single-cell libraries according to the manufacturer's protocol (10X Genomics). Quality control of the libraries was performed using the Agilent BioAnalyzer High Sensitivity Kit. The individual barcoded single-cell RNA-seq libraries were sequenced on the NovaSeq PE150 (Illumina).

### 2.5 Single-cell RNA-seq data analysis

Read alignment and quality control (QC) were performed using the 10x Genomics Cell Ranger (v3.0.2) and the mm10 reference genome. Next, we used the R package Seurat (version 5.0.0) [18] for post-sequencing analyses. The number of genes detected in cells, number of molecules detected, mitochondrial percentage, and ribosomal proportions were used as initial QC filters, and the thresholds were determined by visual inspection. Next, the doublets were removed by doubletFinder [19] using the number of principal components (PCs) determined by PCA elbow plots for each sample. The batch effects were removed using Harmony [20], and the data was later normalized and scaled for further analyses. Dimensionality reduction and Louvain clustering were conducted after choosing the number of PCs based on elbow plots. We next collected markers for monocytes (Lyz2, Itgam, Ly6c1, Ccr2, Cx3cr1), monocytes or neutrophils (Il1b, Csf3r, Ccl8), natural killer/NK cells (Nkg7, Ncr1, Klrk1, Klrb1c), neutrophils (Ccl6, Retnlg, Csf3r, Ly6g, S100a8, Il1r2, Trem1, Ceacam1), pro-B cells (Cd79a, Siglecg, Cd22, Il7r, Cd34, Cd38), pre-B cells (Ebf1, Cd79b, Cd34, Cd38, Cd79a, Mme), CD4 T cells (Ccr2, Il17ra, Cd3d, Cd3e, Cxcr4, Ltb, Trbc2), regulatory T cells (Foxp3, Cd3e, Il2ra, Ctla4), CD8 T cells (Ms4a4b, Trbc2, Tnf, Ifng, Il2, Cxcr3, Tbx21, Il4, Il5, Ccr4, Gata3, Il10, Irf4, Ccr6, Klrb1c, Il17a, Rorc), macrophages (Cd9, Cstb, Itgam, Adgre1, Ccr5), MSC or osteoblastic lineage cells (Col1a1, Fbn1, Cxcl12, Pdgfra, Bglap2, Sparc, Ifitm5), osteoclasts (Ctss, Cd68, Ctsk, Atp6v0d2), endothelial cells (Pecam1, Cdh5, Adamts13, Kdr), proliferating cells (Mki67, Top2a, Pcna, Mcm2), and dendritic cells/DCs (Cd8a, Clec9a, Itgae, Itgax, Thbd, Xcr1, Cd207, Itgam, Notch2, Sirpa) for manual cluster annotation. For clusters not expressing the above markers, we calculated the cluster-specific genes and inspected their cellular abundance by Enrichr webserver. For a better cell type definition granularity, we combined monocytes, monocytes/neutrophils, and neutrophils as “Myeloids”. A total of 13 clusters corresponding to different cell types were finally determined.

Several further analyses were conducted on the annotations. The differentially-expressed genes (DEGs) from key cell types were calculated by FindMarkers function in Seurat and visualized by EnhancedVolcano (https://bioconductor.org/packages/release/bioc/html/EnhancedVolcano.html) package. The ligand or receptor (L/R) lists were collected from CellTalkDB and intersected with DEGs by in-house scripts. DEGs with the absolute value of log2(Fold Change) > 1 and adjusted P-value <0.05 were further selected for Gene Ontology and KEGG pathway enrichment analyses using ClusterProfiler tool [21]. Additionally, we collected pro-inflammatory and anti-inflammatory cytokines [22] and inspected their average expression in immune cells. The AUCell tool [23] was also used for the assessment of the activities of these cytokines in certain cell types. Moreover, markers for T cell exhaustion (Pdcd1, Ctla4, Havcr2, Lag3) were collected to calculate average expression in cells and AUCell activities. Activities of MDSC markers [24] were also compared between macrophages and other myeloid cells. As for unsupervised expression trend identification, the average expression of all genes was calculated for DCs and NK cells regarding their relatively higher abundance in three conditions. Later the average gene expressions were clustered using fuzzy C-means algorithm via TCseq R package (https://www.bioconductor.org/packages/release/bioc/html/TCseq.html). Key genes after unsupervised clustering were further selected for GO and KEGG pathway enrichments. Communications between cells were finally quantified and visualized by CellChat [25]. Only interactions between more than 20 cells were reported. The intersection between key DEGs and L/Rs involved in cell–cell communications was conducted by in-house scripts.

## 3 Results

### 3.1 Differential cell composition revealed by scRNA-seq

**Fig. 1:**
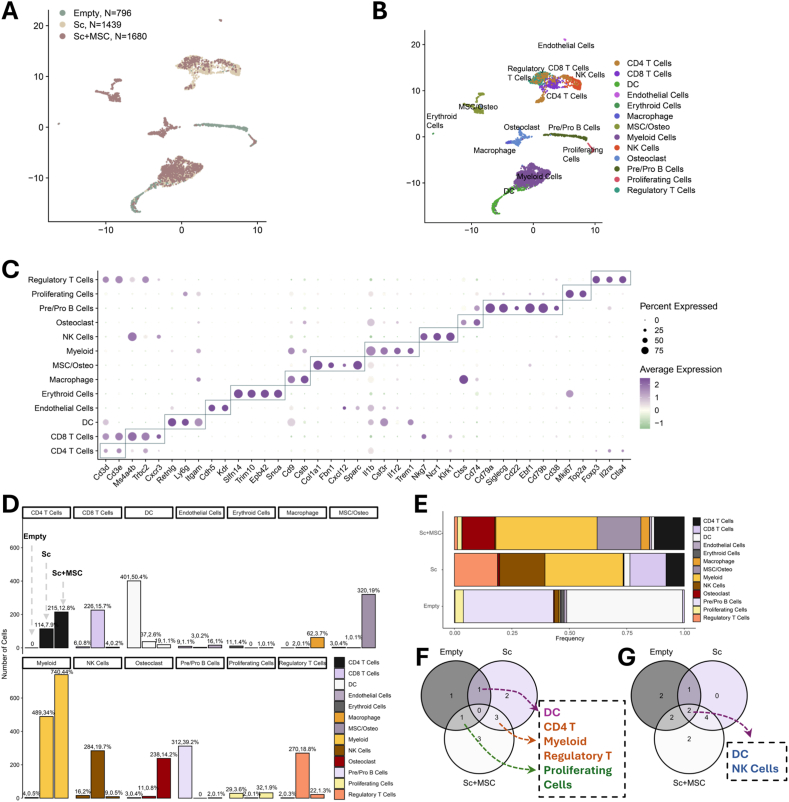
Differential cell composition revealed by scRNA-seq. (A) Uniform manifold approximation and projection (UMAP) plot showing cell distribution of sequenced single cells from three groups (B). Louvain Clustering of the same UMAP plot defined thirteen cell populations by the marker genes in panel C. (C) Dot plot showing marker genes for each cell cluster. (D) Cell numbers of each cell type among three groups. (E) Percentual fraction of cell types per group. (F) Venn plot shows the cell types with more than 20 cells and (G) 5 cells among the three groups.

To highlight the differences in cell heterogeneity among the three experimental groups, we first described the cell distribution using a Uniform Manifold Approximation and Projection (UMAP) plot (Fig. 1A) with quality-filtered cells from all groups. We identified thirteen distinct populations and annotated each cluster using UMAP with Louvain clustering based on marker gene expression (Fig. 1B and C). We then compared the cell compositions from the three conditions (Fig. 1D and E). In the Empty group, the cells were primarily dendritic cells (50.4 %) and B cells (39.2 %). The Sc group showed significant infiltration of myeloid cells (34 %), NK cells (19.7 %), regulatory T cells (18.8 %), CD8 T cells (15.7 %), and CD4 T cells (7.9 %) at the defect site. The Sc + MSC group exhibited major infiltration of myeloid cells (44 %), MSCs and osteoblastic lineage cells (MSC/Osteo) (19 %), osteoclasts (14.2 %), and CD4 T cells (12.8 %) (Fig. 1E). Notably, a key difference between the Sc and Sc + MSC groups was the proportion of various types of immune cells: the Sc + MSC group had more macrophages but fewer NK cells, CD8 T cells, and regulatory T cells compared to the Sc group. Additionally, MSCs and osteoblastic lineage cells (MSC/Osteo), and osteoclasts were almost exclusively found in the Sc + MSC group. In our study, cells from all the mice of each group were pooled instead of barcoding each individual due to the insufficient number of cells per sample for reliable downstream analysis. Pooling samples within each group also allows us to minimize technical variability and batch effects during library preparation and sequencing. To make sure that the trends of the data are consistent across animals, robustness analyses to evaluate the representativeness and stability of the pooled data were conducted. In the random subsampling analysis (Fig. S2B), we randomly sampled 30 % of all the cells 100 times and compared the resulting cell-type distributions with the original full dataset. The subsampled distributions showed minimal deviation from the original, indicating that no single animal dominated the pooled data and that the overall cellular composition remained stable across subsamples. In the marker expression stability assessments in the key cell types (Fig. S2C), we selected a representative dominant cell type (e.g., dendritic cells in the Empty group, myeloid cells in the Sc group, and MSC/osteolineage cells in the Sc + MSC group). We then compared the expression levels of canonical marker genes between the original and the 100x-random subsampled cells (using t-tests). No statistically significant differences were observed, supporting the conclusion that pooling did not distort the underlying transcriptional signatures of key cell types. We further examined the cells detected in all three groups with more than 20 cell counts and 5 cell counts. CD4 T cells, myeloid cells, and regulatory T cells were present at the defect site following scaffold implantation, both with and without MSCs (Fig. 1F). DC and NK cells were present in all three groups (Fig. 1G). We extracted the NK and T cell subsets to check their distribution due to some possible overlap with CD4 T cells (Fig. S3A). Ncr1 (mouse-specific NK cell marker), Cd3d, and Cd3e (T cell markers) were inspected in the extracted clusters. Expectedly, Ncr1 was specifically expressed in NK cells, while the T cell markers demonstrated higher T cell expression specificity (Fig. S3B). Moreover, a significantly higher level of Ncr1 was observed in NK cells, and T cells showed higher Cd3d and Cd3e (Fig. S3C). These results endorsed our annotation result on NK and T cell clusters. We further performed a differential gene expression analysis across the groups based on this observation.

### 3.2 Differential gene expression in major cell populations

**Fig. 2:**
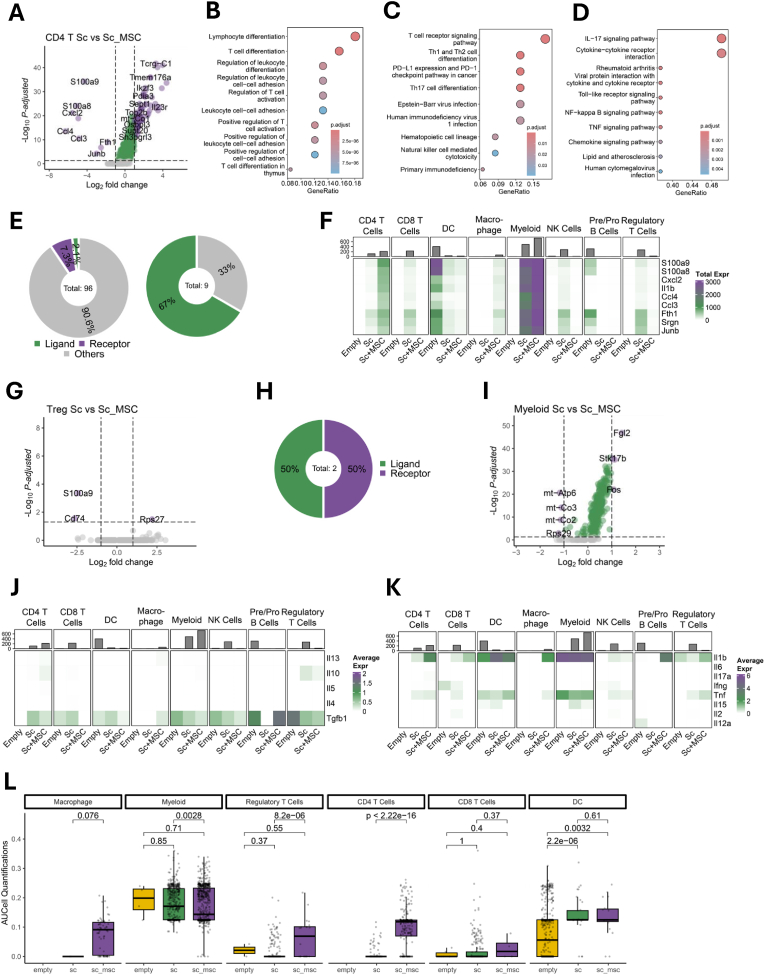
Differential gene expression in major cell populations. (A) Volcano plot showing the differentially expressed genes (DEGs) in CD4 T cells between the Sc and Sc + MSC groups. (B) GO pathway analysis of lowly expressed genes in CD4 T cells from the Sc + MSC group. (C) KEGG pathway analysis of lowly expressed genes in CD4 T cells from the Sc + MSC group. (D) KEGG pathway analysis of highly expressed genes in CD4 T cells from the Sc + MSC group. (E) The proportion of ligands and receptors identified among lowly (left) and highly (right) expressed genes in CD4 T cells from the Sc + MSC group. (F) Heatmap showing expression levels of highly expressed genes in CD4 T cells from the Sc + MSC group across different immune cell types. (G) Volcano plot showing DEG s in Treg cells between the Empty and Sc + MSC groups. (H) Pie chart showing the proportion of ligands and receptors among highly expressed genes in Treg cells from the Sc + MSC group. (I) Volcano plot showing DEGs in Myeloid cells between the Sc and Sc + MSC groups. (J) Heatmap showing expression levels of anti-inflammatory genes across different immune cell types. (K) Heatmap showing expression levels of pro-inflammatory genes across different immune cell types. (L) Pro-inflammatory score using AUCell quantification.

In CD4 T cells, both the Sc and Sc + MSC groups exhibited distinct expression profiles. Notably, *Ccl3*, *Ccl4*, and *Cxcl2* were highly expressed in the Sc + MSC group (Fig. 2A). To further explore the functional implications of genes with reduced expression in the Sc + MSC group, we conducted the Gene Ontology (GO) pathway analysis (Fig. 2B). This analysis identified lymphocyte differentiation and T cell differentiation as the top enriched pathways. Additionally, KEGG pathway analysis highlighted T cell receptor signaling and Th1/Th2 cell differentiation as the top two pathways (Fig. 2C). Notably, Th17 cell differentiation also emerged as one of the top 10 pathways, indicating that MSCs exert a significant influence on T cell differentiation. For the highly expressed genes in the Sc + MSC group, inflammatory-related signaling pathways such as the IL-17 signaling pathway, the NF‐κB signaling pathway, the TNF signaling pathway, and the chemokine signaling pathways were prioritized (Fig. 2D). We further intersected the differentially expressed genes in the Sc + MSC group with known ligands and receptors. Interestingly, 67 % of the upregulated genes in the Sc + MSC group were ligands (Fig. 2E). Intriguingly, we observed that the highly expressed genes in CD4 T cells from the Sc + MSC group were almost exclusively enriched in CD4 T cells, with minimal expression in other immune cell types (Fig. 2F). Similarly, we identified the differentially expressed genes (DEGs) in proliferating cells between the empty and Sc + MSC groups. While most of the DEGs were downregulated, *Cxcl2* and *Il-1b* were notably highly expressed in the Sc + MSC group (Fig. S4A). We further analyzed the GO pathways associated with the downregulated genes and found that the top enriched pathways were related to chromosome regulation and cell cycle regulation (Fig. S4B). Similarly, KEGG pathway analysis prioritized the cell cycle as the top affected pathway (Fig. S4C). We also identified the differentially expressed genes (DEGs) in NK cells between the empty and Sc groups (Fig. S4D). Only S100a8 and Ttc3 were upregulated in the Empty group. We noticed that there is a difference in the NK cell numbers—comparing 16 cells (2 %) in the Empty group to 284 (19 %) in the Sc group (Fig. 1D), we down-sampled NK cells in the Sc group to match the Empty group 100 times for identifying the top genes upregulated in the Empty group to verify the findings. S100a8 and Ttc3 were demonstrated as the genes with top 2 occurrences in 100 iterations (Fig. S5A), denoting the reliability of our DEG analysis. Additionally, we applied the MAST tool [26] to identify DEGs between the two NK cell groups, as it incorporates a generalized linear model to adjust for cell cluster number variability. S100a8 and Ttc3 were expected to be among the top 10 genes significantly upregulated in the Empty group as identified by MAST (Fig. S5B). When intersecting results from the three methods, S100a8 and Ttc3 were the only overlapping genes (Fig. S5C). Their expression also showed clear group-specific patterns (Fig. S5D). These results demonstrate the high confidence of our DEG analysis on NK cells. We further identified the DEGs in DCs between the Empty and Sc groups (Fig. S4E). GO analysis showed enriched pathways of upregulated genes in the Empty group related to Neutrophil mediated immunity, neutrophil activation, and granulocyte activation (Fig. S4F) while KEEG analysis revealed the Neutrophil extracellular trap formation (Fig. S4G). Meanwhile, GO analysis showed the upregulated genes in the Sc group were associated with leukocyte-related activities, and regulation of myeloid cell differentiation (Fig. S4H). KEEG pathway analysis showed only the ferroptosis pathway was associated with the upregulated genes in the Sc group.

As for DEGs from regulatory T cells (Tregs), *S100a9* and *Cd74* were upregulated in the Sc + MSC group, while *Rps27* was downregulated (Fig. 2G). Notably, one of the highly expressed genes (*S100a9*) encodes a ligand, while another (*Cd74*) encodes a receptor (Fig. 2H). Additionally, we analyzed DEGs within the myeloid cell cluster between the Sc and Sc + MSC groups (Fig. 2I). *Fgl2*, a key mediator gene of inflammation, was downregulated in the Sc + MSC group. Thus, we investigated the expression of both anti-inflammatory factors (*Il13, Il10, Il5, Il4, Tgfb1*) and pro-inflammatory factors (*Il1b, Il6, Il17a, Ifng, Tnf, Il15, Il2, Il12a*) in various immune cell types across the three groups. In the Sc + MSC group, *Tgfb1* levels were consistently decreased across most immune cell types (Fig. 2J), while *Il1b* was increased in T cells, macrophages, and pre/pro B cells (Fig. 2K). To integrally quantify the expression levels of pro-inflammatory and anti-inflammatory factors, we employed AUCell analysis, which exhibited similar trends with a significantly elevated pro-inflammatory level in Tregs and CD4 T cells and a higher trend in macrophages in the Sc + MSC group compared with the Sc group (Fig. 2L). Limited changes were observed in the anti-inflammatory levels between these two groups (Fig. S4I). We further analyzed the expression levels of genes associated with T cell exhaustion, including *Pdcd1*, *Ctla4*, *Havcr2*, and *Lag3*, in T cell populations (Fig. S4J). Our analysis revealed that *Ctla4* expression was elevated in both CD4 and CD8 T cells in the Sc + MSC group compared to the Sc group. *Pdcd1* was decreased in CD4 and CD8 T cells in the Sc + MSC group. Similar results were observed when employing the AUCell tool (Fig. S4K). Moreover, myeloid-derived suppressor cells (MDSCs), a subset of myeloid cells with immunosuppressive functions [24], were enriched in the Sc + MSC group, as demonstrated by AUCell quantification (Fig. S4L). These findings suggest that MSCs can contribute to the formation of an immunosuppression microenvironment by enhancing T cell exhaustion markers and recruiting immunosuppressive myeloid populations.

### 3.3 The heterogeneity of DC & NK cells in different groups

**Fig. 3:**
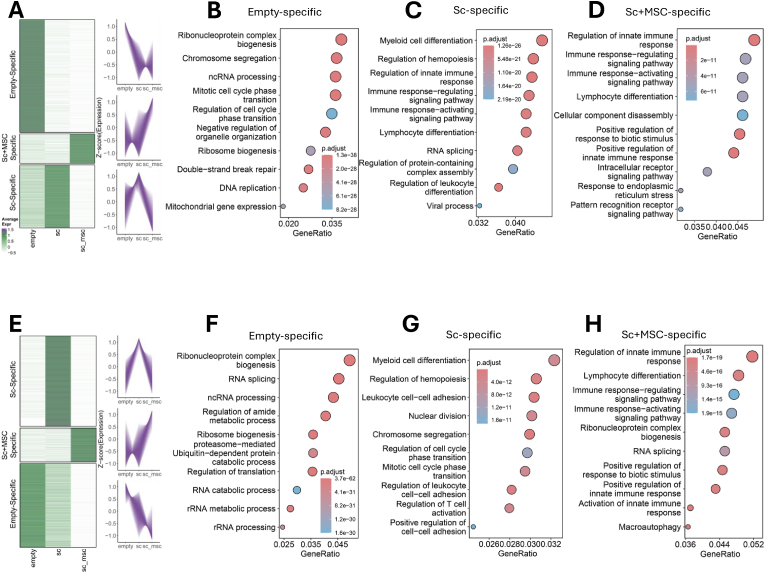
The heterogeneity of DC & NK cells in different groups. (A) Clustering analysis results on DCs using TCseq (Time Course sequencing data analysis) tool. GO pathway analysis on the first cluster (B) Empty-specific genes, (C) Sc-specific genes, and (D) Sc + MSC-specific genes of DCs. (E) Clustering analysis results on NK cells using TCseq tool. GO pathway analysis on the first cluster (F) Empty-specific genes, (G) Sc-specific genes, and (H) Sc + MSC-specific genes of NK cells.

Given the observation of DCs and NK cells in all the groups and their roles in the complex immune regulation required for successful bone repair, we further investigated their sequential expression trends using the TCseq tool. Our analysis revealed three distinct subsets of genes demonstrating different expression patterns in DCs, namely Empty-specific, Sc + MSC-specific, and Sc-specific groups (Fig. 3A). We performed GO (Fig. 3B–D) and KEGG (Fig. S6A–C) pathway analyses for Empty-specific, Sc-specific, and Sc + MSC-specific genes. The Empty-specific genes are associated with ribonucleoprotein complex biogenesis, chromosome segregation, ncRNA processing, and cell cycle regulation (Fig. 3B). In contrast, the Sc-specific genes are linked to myeloid cell differentiation, regulation of hemopoiesis, and immune response modulation (Fig. 3C). The Sc + MSC-specific genes, more specifically, are associated with the regulation of innate immune response, immune response modulation, and activation of signaling pathways (Fig. 3D). These results suggest that MSCs exert immunomodulatory effects on DCs, thereby influencing immune responses and potentially enhancing bone healing. Similarly, TCseq analysis of the NK cells identified three gene sets with distinct expression patterns among the three experimental groups (Fig. 3E). GO (Fig. 3F–H) and KEGG (Fig. S6D–F) pathway analyses were conducted for the Empty-specific genes, Sc-specific genes, and Sc + MSC-specific genes. Similar to the results in DCs, the Empty-specific genes are associated with ribonucleoprotein complex biogenesis, RNA splicing, and ncRNA processing (Fig. 3F). The Sc-specific genes are linked to myeloid cell differentiation, regulation of hemopoiesis, and leukocyte cell–cell adhesion (Fig. 3G). Notably, GO analysis of the Sc + MSC-specific genes from NK cells highlighted similar top pathways as those observed in DCs, which were more specifically involved in immunomodulation, including the regulation of innate immune response, lymphocyte differentiation, immune response modulation, and activation of signaling pathways (Fig. 3H). These findings indicate that, compared to the Empty group, implantation in the Sc and Sc + MSC groups significantly altered local immune responses, potentially enhancing the bone regeneration process. The introduced MSCs in the Sc + MSC group had a further significant impact on various immune cell types, orchestrating adaptive immune responses and providing essential signals for immune cell differentiation and coordination during bone repair.

### 3.4 Cell–cell communications between major cell populations in different groups

**Fig. 4:**
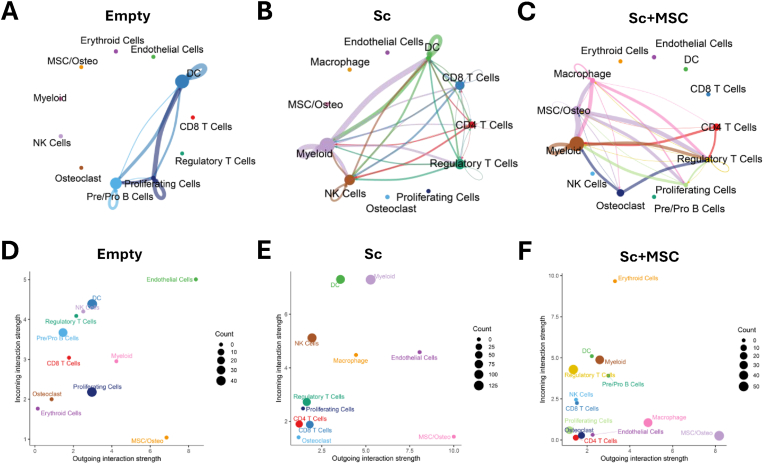
Cell–cell communications between major cell populations in different groups. Circos plots showing potential cell–cell interactions among different cell types in the (A) Empty, (B) Sc, and (C) Sc + MSC groups, as predicted by CellChat. Node size indicates the number of interactions for each cell type, while edge width represents the number of significant ligand–receptor pairs between two cell types. Scatter plots showing outgoing interaction strength (ligand) versus incoming interaction strength (receptor) for the (D) Empty, (E) Sc, and (F) Sc + MSC groups. Node size represents the number of interactions.

Understanding the interaction between MSCs and immune cells during bone healing is crucial for unraveling the mechanisms behind bone regeneration and the therapeutic potential of MSC-based treatments. To explore these interactions, we used CellChat (Method) to identify ligand–receptor pairs across the major cell types depicted in the previous results. The circos plots revealed broadcast ligands and extensive communication between cognate receptors in different conditions (Fig. 4A–C). Notably, the Sc and Sc + MSC groups exhibited a greater number of interactions between different cell types, whereas the empty group showed fewer interactions, primarily between DCs, pre/pro B cells, and proliferating cells. Additionally, cell–cell communications between the Sc and Sc + MSC groups exhibited certain experimental specificity. In the Sc group, the majority of interactions were observed among various T cells, NK cells, and myeloid cells. In contrast, the Sc + MSC group demonstrated more interactions between macrophages, MSCs/osteoblasts, and T cells. To investigate the functional roles of each cluster in the three groups, we assessed whether clusters primarily act as signal senders or receivers (Fig. 4D–F). Our analysis revealed that the MSC/Osteo clusters in all three groups predominantly function as signal senders rather than receivers. Macrophages in the Sc group displayed a balanced role as both senders and receivers, whereas macrophages in the Sc + MSC group were predominantly signal senders. This finding suggests that MSCs influence how immune cells, such as macrophages, interact with other cell types. To summarize the interactions among different clusters within each group, we generated dot plots illustrating these interactions (Fig. S7). Additionally, we examined whether specific upregulated genes in the three groups contributed to cell–cell communications (Fig. S7D). In the Empty group, the upregulated genes were primarily associated with interactions between proliferating cells and B cells or DCs. In the Sc group, the upregulated genes encoded receptors were involved in the interactions between myeloid cells and CD4 T cells. Notably, in the Sc + MSC group, the upregulated genes participated in a broader range of interactions, primarily between CD4 T cells, MSC/Osteo, macrophages, and proliferating cells with regulatory T cells or myeloid cells. Furthermore, interactions were observed between MSC/Osteo clusters or macrophages with regulatory T cells. These findings highlight the diverse cellular interactions mediated by MSCs, demonstrating their influence on the communication dynamics between immune and stromal cell populations within the bone healing microenvironment.

## 4 Discussion and conclusion

Stem cell therapies harness the unique ability of multipotential cells to differentiate into various cell types and self-renew, making them powerful tools for tissue repair and treatment of disease. MSC-based therapies have recently garnered significant attention. The Food and Drug Administration (FDA) approved the first MSC therapy (remestemcel-L-rknd from Ryoncil, Mesoblast, Inc.) to treat steroid-refractory acute graft-versus-host disease on December 18, 2024. China's top drug regulator, the National Medical Products Administration (NMPA), has recently approved the nation's first MSC therapy (hUC-MSC PLEB001 from Platinum Life Excellence Biotech) to treat acute graft-versus-host disease for patients aged 14 and above. These approvals pave the way for using MSCs in treating serious diseases and hopefully, in the future, enhancing tissue regeneration.

Critical-sized bone defects cannot heal spontaneously without appropriate surgical interventions, often leading to nonunion or delayed union. Efforts have been made to explore stem cell transplantation to address bone defects. MSCs play a crucial role in bone healing [27]. Implanting exogenous MSCs using a bioengineered scaffold is a common approach for treating large segmental bone defects in preclinical models [28,29]. Previously we showed enhanced healing of long bone defects using MSC implantation in the μRB scaffold locally immediately after creating the defect [11]. However, this strategy showed limited efficacy in healing established nonunions of critical-size long bone defects (Fig. S1A–C) in our established animal model [16]. In the current study, secondary surgeries were performed four weeks after the generation of the defect when the defect had transitioned from the acute inflammatory phase to a chronic, non-healing scenario. This time point is critical for modeling the clinical condition of the chronic inflammatory stage of a nonunion, as a therapy to achieve union at this stage is an unmet clinical need.

Autogenous bone grafting is the most common clinical treatment for critical-size bone defects, as it is a source of MSCs and other cells relevant to bone healing. Niche cells, including inflammatory cells like macrophages and lymphocytes, create a specific microenvironment that orchestrates the MSC response in different scenarios. Changes in this microenvironment, such as the recruitment of immune cells to the injury site, are crucial for initiating fracture healing [30] and stimulating stem cells to differentiate into osteoblasts, facilitating tissue repair [31]. Interactions between MSCs and local cells are critical to the activation of MSCs and provide important cues to guide their phenotype [9]. However, the cellular dynamics after the MSC implantation are still largely unknown, especially in the scenario of critical-sized defects.

We observed that the MSC/Osteo was significantly higher in the Sc + MSC group, which is expected since we embedded MSCs into the scaffolds. A similar observation was confirmed in the same model using mass cytometry. Notably, we found the majority (72 %) of the MSCs in the defect site were recruited from the host mice [16]. This observation verified the current creation of MSC-based therapy in the current study. The implantation of exogenous MSCs could further facilitate the recruitment of endogenous MSCs and other cells relevant to bone healing and therefore has the potential to enhance local bone regeneration.

In the Sc + MSC group, important interactions between MSC/Osteo and macrophages, CD4 T cells, Tregs, and myeloid cells were observed. Further investigations revealed that MSC/Osteo provided higher outgoing interaction strength compared to other cells, indicating their critical role in modulating the microenvironment of the defect site (Fig. 4F). These MSCs are actively involved in recruiting and modulating other cell types to orchestrate a coordinated response that is necessary for fracture healing, including resolution of inflammation, angiogenesis and bone regeneration [32,33]. During fracture healing, macrophages play a key role in clearing debris and releasing cytokines and chemokines that regulate healing. We found more cell–cell interactions between MSCs and macrophages in the Sc + MSC group (Fig. 4C). Interestingly, Thbs1/2-CD36 signaling was observed in the Sc + MSC group, driving macrophages toward an M2 anti-inflammatory phenotype, which is crucial for resolving inflammation and transitioning to the repair phase [34]. Thbs1/CD36 signaling also promotes wound resolution by coordinating macrophage efferocytosis for bone deposition [35]. These signaling pathways can also support macrophage M1 to M2 polarization [34], promoting the transition from the inflammatory to the repair phase. Other studies have shown the important role of macrophages in bone defect regeneration in vitro and *in vivo* [36,37]. We also identified the roles of *Col6a1/2/3* and *Col1a1/Col1a2* to CD44. Interactions of CD44 with hyaluronic acid are key factors in the interaction between T cells and MSCs, inducing MSC migration to injured tissues [38].

In addition to modulating macrophages, the role of T cell subtypes in critical-sized bone defects is highlighted by the differences in T cell populations found in this study. A previous study using the MSC membrane-coated μRB scaffold in critical-sized cranial bone defects showed that it induced apoptosis of CD8 T cells and enhanced the differentiation of regulatory T cells, in addition to macrophage polarization [39]. Our results also showed differences in CD8 T cells and regulatory T cells in the μRB scaffold with and without MSCs. More CD4 T cells were found in the Sc + MSC group (Fig. 2D), indicating the critical role of CD4 T cells in MSC therapy. It has been reported that new bone formation at the implantation site with titanium implants was significantly lower in T cell-deficient mice compared to T cell-competent mice, with the absence of CD4 T cells exacerbating these effects more than the absence of CD8 T cells [40]. Our DEG analysis further identified and confirmed lymphocyte differentiation and T cell-related processes, including T cell differentiation and regulation of T cell activation. KEGG analysis also confirmed T cell receptor signaling, Th1/Th2 cell differentiation, and Th17 cell differentiation as downregulated pathways in the Sc + MSC group (Fig. 3C). MSCs can promote the generation and function of Th2 cells and Tregs while inhibiting Th1 and Th17 cells [41]. The changes in T cells and the interaction between MSCs and T cells are critical for bone regeneration. The recruitment of CD4 T cells and modulation of Th1/Th2 polarization by manganese-doped silicon-hydroxyapatite nanowires distinctly fostered the osteogenesis of bone marrow stromal cells and enhanced the healing of a mandibular bone defect. This immune milieu also elevated IL-4, secreted by Th2 cells, which is critical for Th2 cell development [42]. IL-4, which can polarize M1 macrophages to the anti-inflammatory M2 phenotype, is a promising target for enhancing bone defect healing [14]. Our previous studies showed that genetically modified MSCs overexpressing IL-4 could further enhance the healing of long bone critical-sized defects after the acute inflammation stage [11,12]. Our results also highlighted the IL-17 signaling pathway in the Sc + MSC group (Fig. 2D). Targeting the IL-17 pathway is a potential approach for various musculoskeletal disorders [43]. The pro-inflammatory cytokine IL-17 stimulates osteoclastogenesis and osteoclast-induced bone loss. However, the role of IL-17 in bone regeneration is still controversial. It has been reported that IL-17 stimulated osteoblast differentiation, mineralization, proliferation, motility, and osteoblast-dependent osteoclastogenesis of in vitro and augmented bone regeneration in a murine critical-size calvarial defect model [44]. The findings of DEGs and pathway analysis in T cells of the Sc + MSCs group (Fig. 2) also suggested that MSCs can contribute to the formation of an immunosuppression microenvironment by interacting with T cells. Additionally, the findings in DCs and NK cells in all three groups (Fig. 3) further underscore the significant impact of MSCs in the Sc + MSC group on multiple immune cell types, modulating the inflammatory microenvironment of the defect site.

The balance between pro-inflammatory and anti-inflammatory immune cells and factors significantly influences the osteogenic potential of MSCs and bone regeneration [45,46]. Compared with the Sc group, the presence of MSCs in the scaffold at the defect site showed its advantage in enhancing defect healing, however, challenges persist. Inflammatory-related pathways, including the NF‐κB signaling pathway, TNF signaling pathway, and chemokine signaling pathway, were observed upregulated in the Sc + MSC groups (Fig. 2D). IL-17 can trigger the NF-κB signaling pathway, resulting in the release of pro-inflammatory and chemotactic factors [47], which was also found in our results. The pro-inflammatory cytokines and pathways have complex roles during bone regeneration. Inflammation is the critical step to initiate bone regeneration, however, failure of a successful resolution of inflammation impairs bone healing [48]. The increased pro-inflammatory factor in the major cell populations, including increased IL-1β and TNF (Fig. 2K) and the higher score (Fig. 2L), further indicated the inflammatory microenvironment in the Sc + MSC. Meanwhile, the depletion of CD4 T cells in the Sc + MSC group was significantly higher than in the other groups. The depletion of CD4 and CD8 T cells was reported to enhance the osteoclast-like cell formation, which impairs bone formation during the regeneration process [49]. The Myeloid-derived suppressor cells (MDSCs) are regulators of the immune system and expand during various pathological conditions, including inflammation [50]. The elevated MDSC level in Sc + MSCs (Fig. S2L) further confirmed the inflammatory microenvironment in the Sc + MSC group. These findings may explain the limited healing during the chronic stage of the nonunion of critical-size bone defects.

In this study, we employed scRNA-seq to investigate the cellular dynamics of MSC-based therapy in a model of long bone critical-sized defects [51] in mice. Limitations to the current study must be recognized. The spatial resolution is an important consideration in bone defect healing, such as the changes between the central region and the proximal or distal ends, however, due to the tiny size of the defect regain and the limited number of cells present in each subregion, we just pooled, minced and dissociated the collected tissues in each group. We also acknowledge that a longitudinal analysis across multiple time points would yield deeper insights into the progression of healing and the evolving cellular landscape. The current study focuses on the early time point following the secondary surgery to capture the early cellular and transcriptional responses to MSC-based therapy and scaffold implantation to provide a high-resolution snapshot of the early events. Future studies integrating spatial transcriptomics or region-specific dissection map cell fate and interactions over time could provide a more comprehensive picture of the cellular dynamics in MSC-based therapy.

The current study generated a high-resolution transcriptional atlas, aiming to explore underlying mechanisms and establish a foundational framework to inform future research. While we fully recognize the importance of validating transcriptomic findings through orthogonal approaches, including immunohistochemistry and flow cytometry, several key considerations limited the inclusion of these analyses in the current study. Firstly, many of the critical cell subsets identified in our study are rare and exhibit subtle but functionally important transcriptional changes. These differences are often beyond the detection capabilities of immunohistochemistry or flow cytometry, particularly given the small tissue samples collected in this study. Secondly, while immunohistochemistry and flow cytometry provide valuable static information on cell presence and marker expression, they are inherently limited in capturing dynamic cellular transitions and intercellular communication. For example, our scRNA-seq analysis revealed that MSCs exhibited markedly higher outgoing interaction strength compared to other cell types, suggesting a central role in modulating the defect microenvironment rather than direct differentiation—an insight that cannot be readily obtained or validated through immunohistochemistry or flow cytometry. Furthermore, although we previously analyzed cell profiles of bone graft using mass cytometry [16], adding this positive control to the current study would provide additional pathway analysis for the “gold standard”. However, mass cytometry relies on a predefined marker panel, which may miss rare or previously uncharacterized populations and cannot fully resolve the dynamic cell–cell communication patterns uncovered by scRNA-seq. These limitations underscore the unique value of scRNA-seq in this study and highlight its role in laying the groundwork for future functional and translational studies. Future studies —particularly those aimed at optimizing MSC- or cell-based therapies for robust bone regeneration—will be more appropriately designed to incorporate validation methods such as flow cytometry and immunohistochemistry to further substantiate and expand upon the current findings.

In this study, we identified the cellular dynamics of MSC-encapsulated scaffolds. MSC implantation created a different immune microenvironment that tended to initiate and enhance the healing process. However, the inflammatory microenvironment may limit the healing of critical-sized bone defects. We highlighted the important cellular crosstalk by MSCs and immune cells, including T cells and macrophages. We also observed a key role of T cells in the defect sites during this early stage. Optimizing this cellular crosstalk early in the healing process could potentially augment MSC-based therapies for subsequent bone regeneration of critical-size bone defects.

## Author contributions

N.Z., S.B.G. conceived the project, N.Z., X.L., and E.H. performed the in vitro and *in vivo* experiments. N. S. and F.Y. supported the scaffold materials, J.Y., Y.W., and S.C. performed the data analysis. N.Z, J.Y., and X.L. wrote the draft manuscript. N.S., Q.G., F.Y., S.K.H.C., and S. B.G. reviewed, edited, and formed the manuscript. All authors approved the final version of the manuscript.

## Data Availability

The raw data supporting the conclusions of this article will be made available by the authors. Requests to access the datasets should be directed to Dr. Ning Zhang at ningzhang@cuhk.edu.hk or Dr. Xueping Li at xuepingli91@gmail.com.

## Fundings

This work was supported by the 10.13039/100000002NIH grants R01AR073145 and R01AR063717 from 10.13039/100000069NIAMS, the Ellenburg Chair in Surgery at 10.13039/100005492Stanford University, and the 10.13039/501100004853CUHK direct grant for research 399871790 and 488036076.

## Declaration of competing interest

The authors declare that they have no known competing financial interests or personal relationships that could have appeared to influence the work reported in this paper.
