# Supplementary material for: Single-cell RNA sequencing reveals early cell dynamics of MSC-based therapy in long bone critical-size defects in mice
Source: J Orthop Translat. 2025 Sep 9;55:121–31. doi: 10.1016/j.jot.2025.08.007 (PMC12454273; doi:10.1016/j.jot.2025.08.007)
Supplement: Multimedia component 1 [file mmc1.pdf]

Figure S1

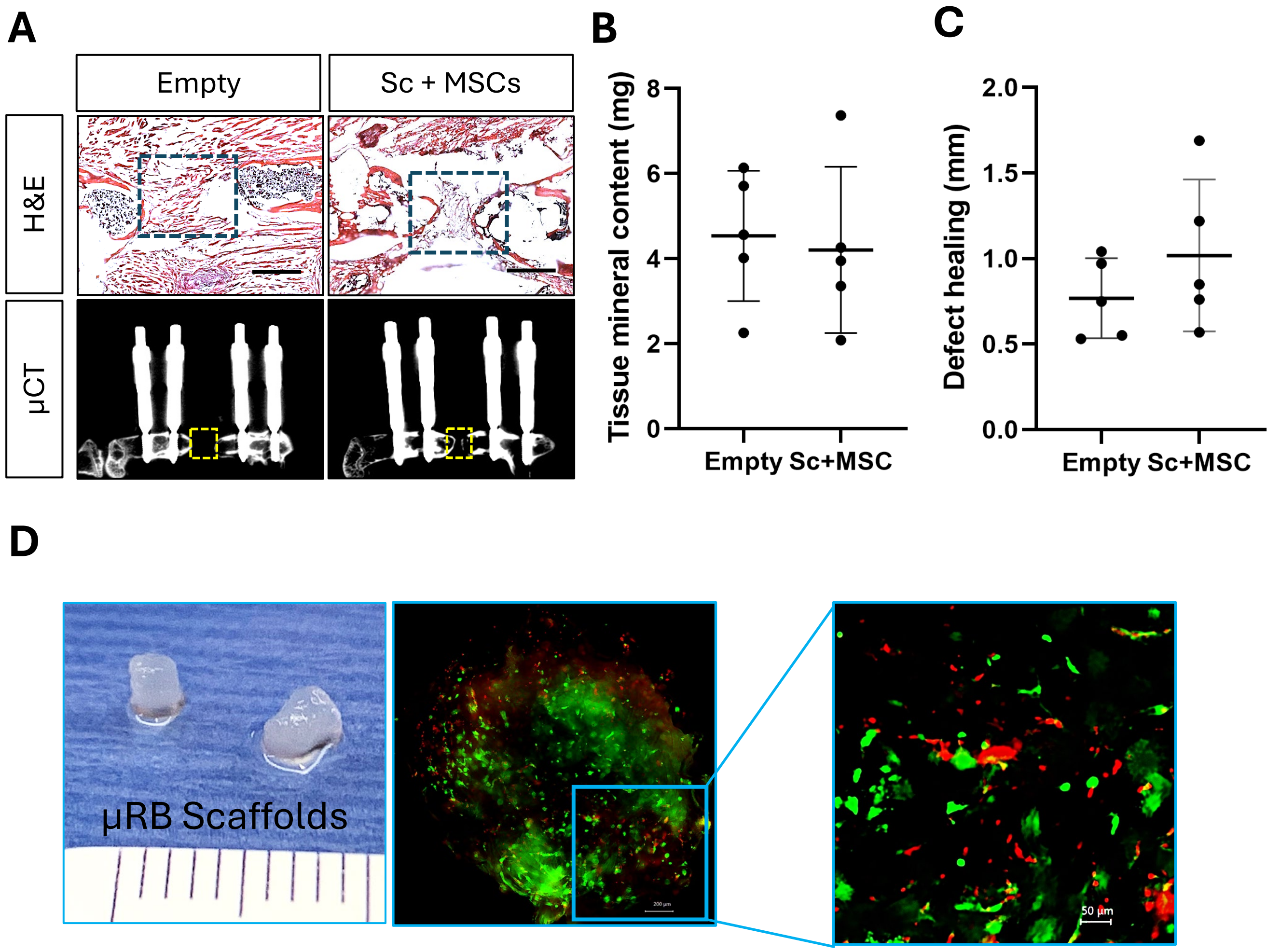

Figure S1. (A) H&E stain and μCT representative images of critical-size bone defect healing 6 weeks after the implantation. (B) Tissue mineral content (mg) and (C) defect healing (mm) of the bone defect area 6 weeks after the implantation. (D) μRB Scaffolds and the MSCs encapsulated in the scaffold, cell viability of 88.96 %±7.77 % after 1 day of culture in vitro.

Figure S2

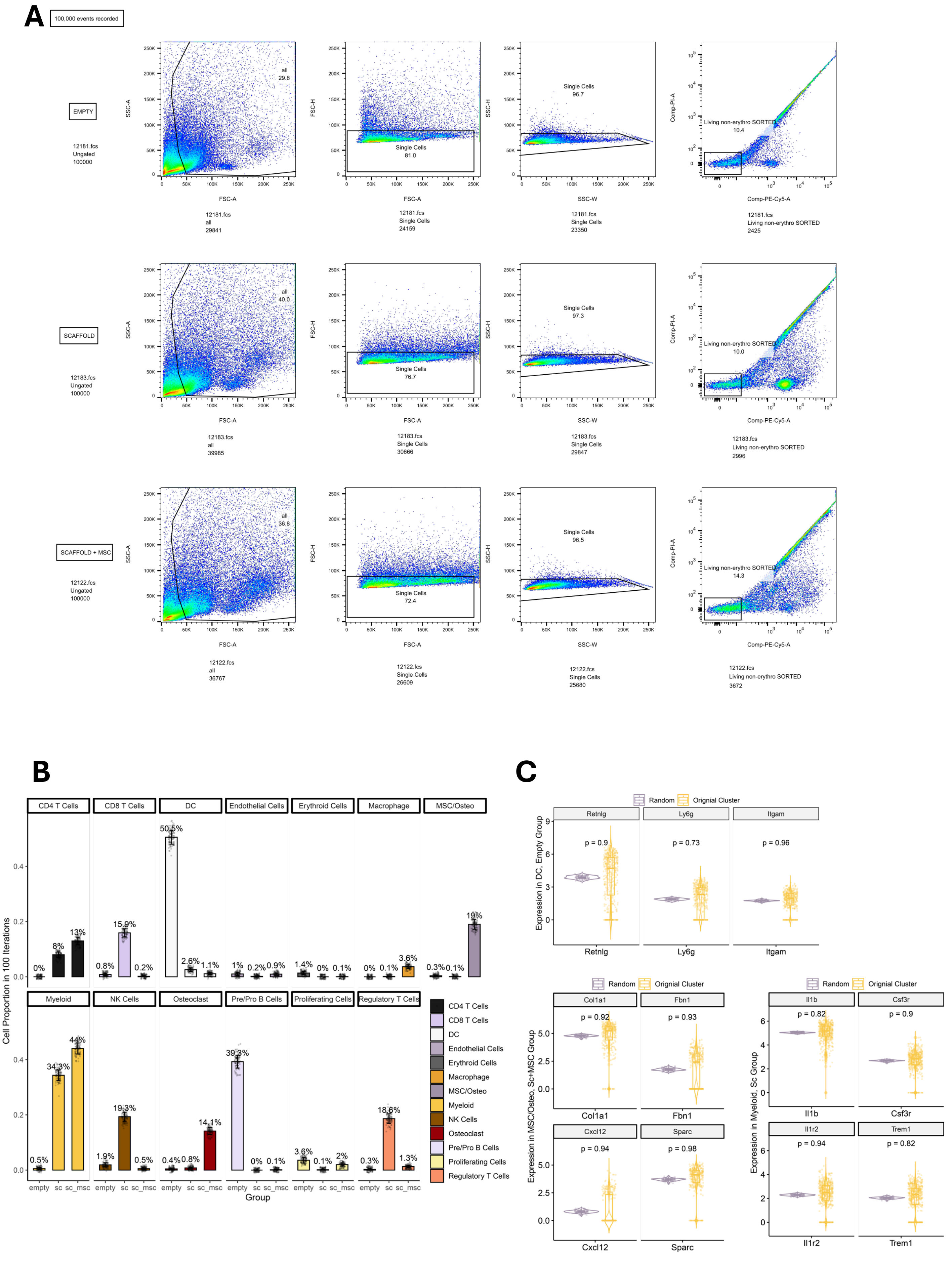

A
100,000 events recorded
EMPTY
12181.fcs
Ungated
100000
SCAFFOLD
12183.fcs
Ungated
100000
SCAFFOLD + MSC
12122.fcs
Ungated
100000
all
29.8
all
40.0
all
36.8
Single Cells
81.0
Single Cells
76.7
Single Cells
72.4
Single Cells
96.7
Single Cells
97.3
Single Cells
96.5
Living non-erythro SORTED
10.4
Living non-erythro SORTED
10.0
Living non-erythro SORTED
14.3
SSC-A
FSC-A
FSC-H
SSC-H
SSC-W
Comp-PI-A
Comp-PE-Cy5-A
12181.fcs
all
29841
12181.fcs
Single Cells
24159
12181.fcs
Single Cells
23350
12181.fcs
Living non-erythro SORTED
2425
12183.fcs
all
39985
12183.fcs
Single Cells
30666
12183.fcs
Single Cells
29847
12183.fcs
Living non-erythro SORTED
2996
12122.fcs
all
36767
12122.fcs
Single Cells
26609
12122.fcs
Single Cells
25680
12122.fcs
Living non-erythro SORTED
3672
B
CD4 T Cells
CD8 T Cells
DC
Endothelial Cells
Erythroid Cells
Macrophage
MSC/Osteo
Myeloid
NK Cells
Osteoclast
Pre/Pro B Cells
Proliferating Cells
Regulatory T Cells
Cell Proportion in 100 Iterations
Group
empty sc sc_msc
C
Random
Orignial Cluster
Retnlg
Ly6g
Itgam
p = 0.9
p = 0.73
p = 0.96
Expression in DC, Empty Group
Col1a1
Fbn1
Cxcl12
Sparc
p = 0.92
p = 0.93
p = 0.94
p = 0.98
Expression in MSC/Osteo, Sc+MSC Group
Il1b
Csf3r
Il1r2
Trem1
p = 0.82
p = 0.9
p = 0.94
p = 0.82
Expression in Myeloid, Sc Group

Figure S2. (A) Flow cytometry gating strategy. For each group (Empty, Scaffold, Scaffold+MSC), single-cell suspensions were first gated to exclude debris and doublets using FSC-A vs. SSC-A and FSC-A vs. FSC-H plots. Singlet events were further gated using SSC-H vs. SSC-W to remove aggregates. Cells were then stained with Propidium Iodide (PI) to identify dead cells and PE-conjugated antibody to label erythroid lineage cells. Live, non-erythroid cells (PI^-, PE^-) were sorted and used for downstream single-cell library preparation. The validation of cell pooling robustness using random subsampling and marker expression analysis is shown in (B) Random subsampling analysis of cell-type composition. 30% of all cells were randomly sampled 100 times. The resulting cell-type distributions were compared with the original full dataset. (C) Marker stability testing in dominant cell types. For each group, a representative dominant cell type was selected (dendritic cells in Empty, myeloid cells in Scaffold, and MSC/osteolineage cells in Scaffold+MSC). Marker gene expression in 100 subsampled subsets (each comprising 30% of cells) was compared to the original cell population using t-tests.

Figure S3

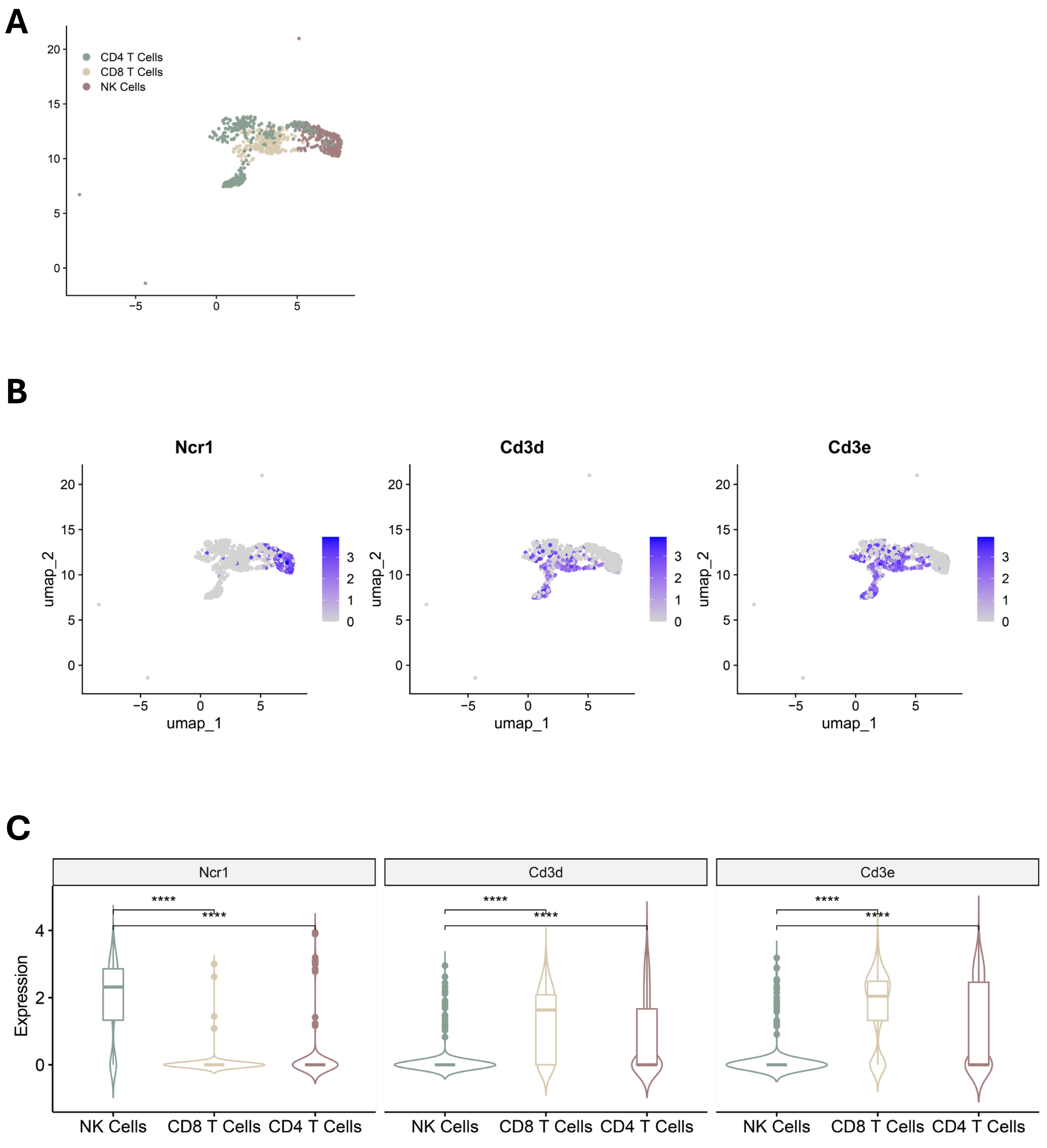

Figure S3. (A) NK and T cell subsets visualized by UMAP. (B) Ncr1, Cd3d, and Cd3e expression in NK and T cell subsets. (C) Expression levels of Ncr1, Cd3d, and Cd3e in NK cells, CD8 and CD4 T cells. (B) Random subsampling analysis of cell-type composition. 30% of all cells were randomly sampled 100 times. The resulting cell-type distributions were compared with the original full dataset.

Figure S4

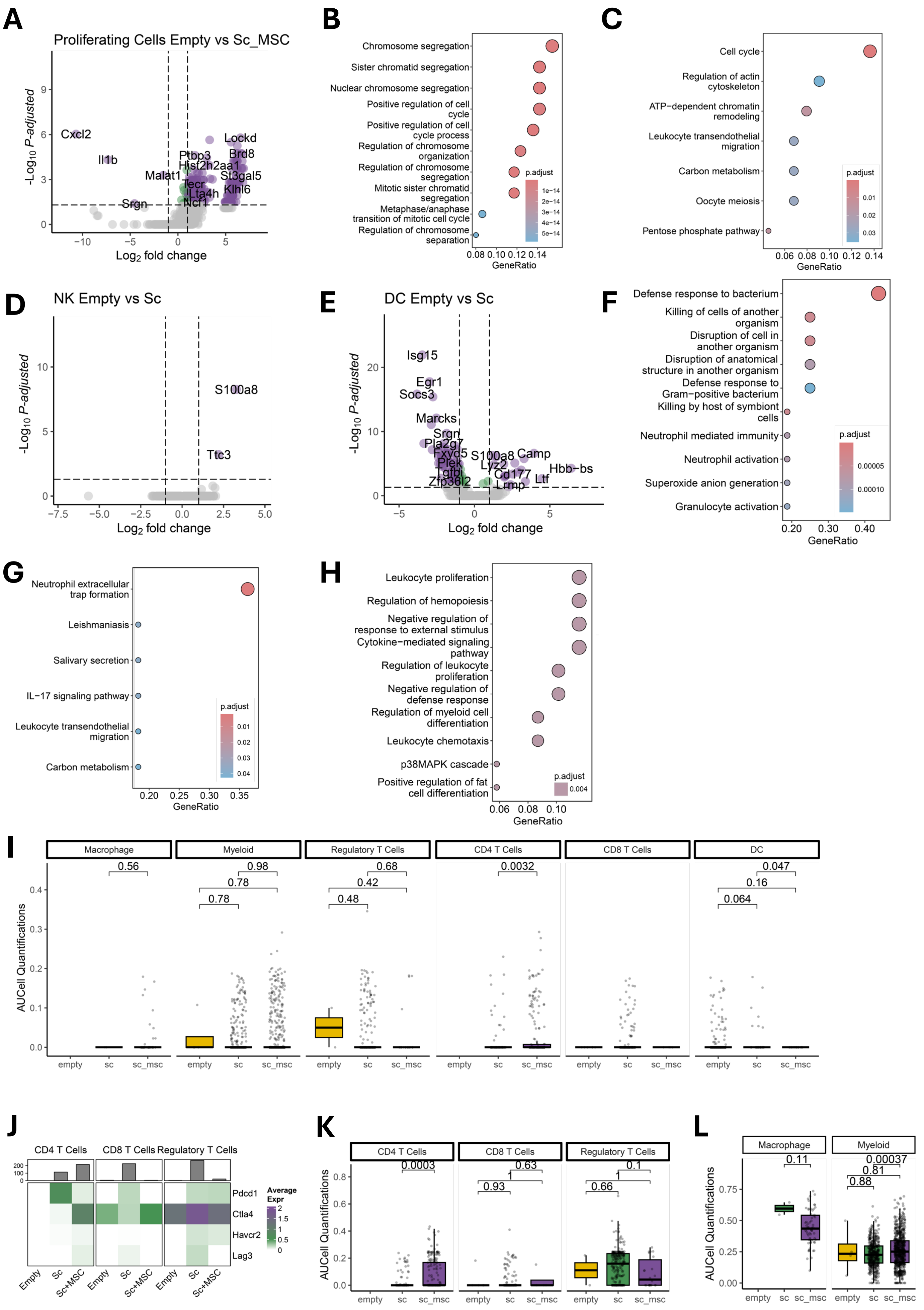

A
Proliferating Cells Empty vs Sc_MSC
B
C
D
NK Empty vs Sc
E
DC Empty vs Sc
F
G
H
I
Macrophage
Myeloid
Regulatory T Cells
CD4 T Cells
CD8 T Cells
DC
AUCell Quantifications
J
K
L

Figure S4. (A) Volcano plot showing the differentially expressed genes (DEGs) in proliferating cells between the Empty and Sc+MSC groups. (B) GO pathway analysis of lowly expressed genes in proliferating cells from the Sc+MSC group. (C) KEGG pathway analysis of lowly expressed genes in proliferating cells from the Sc+MSC group. (D) Volcano plot showing the DEGs in NK cells between the Empty and Sc groups. (E) Volcano plot showing the DEGs in DCs between the Empty and Sc groups. (F) GO pathway analysis of highly expressed genes in the Empty group. (G) KEEG pathway analysis of highly expressed genes in the Empty group. (H) GO pathway analysis of highly expressed genes in the Sc group. KEEG pathway analysis showed that the upregulated genes in the Sc group were only associated with the ferroptosis pathway (data not shown in the figure). (I) Anti-inflammatory score using AUCell quantification. (J) Heatmap showing the expression levels of genes associated with T cell exhaustion. (K) T cell exhaustion using AUCell quantification. (L) MDSC characteristic score using AUCell quantification.

Figure S5

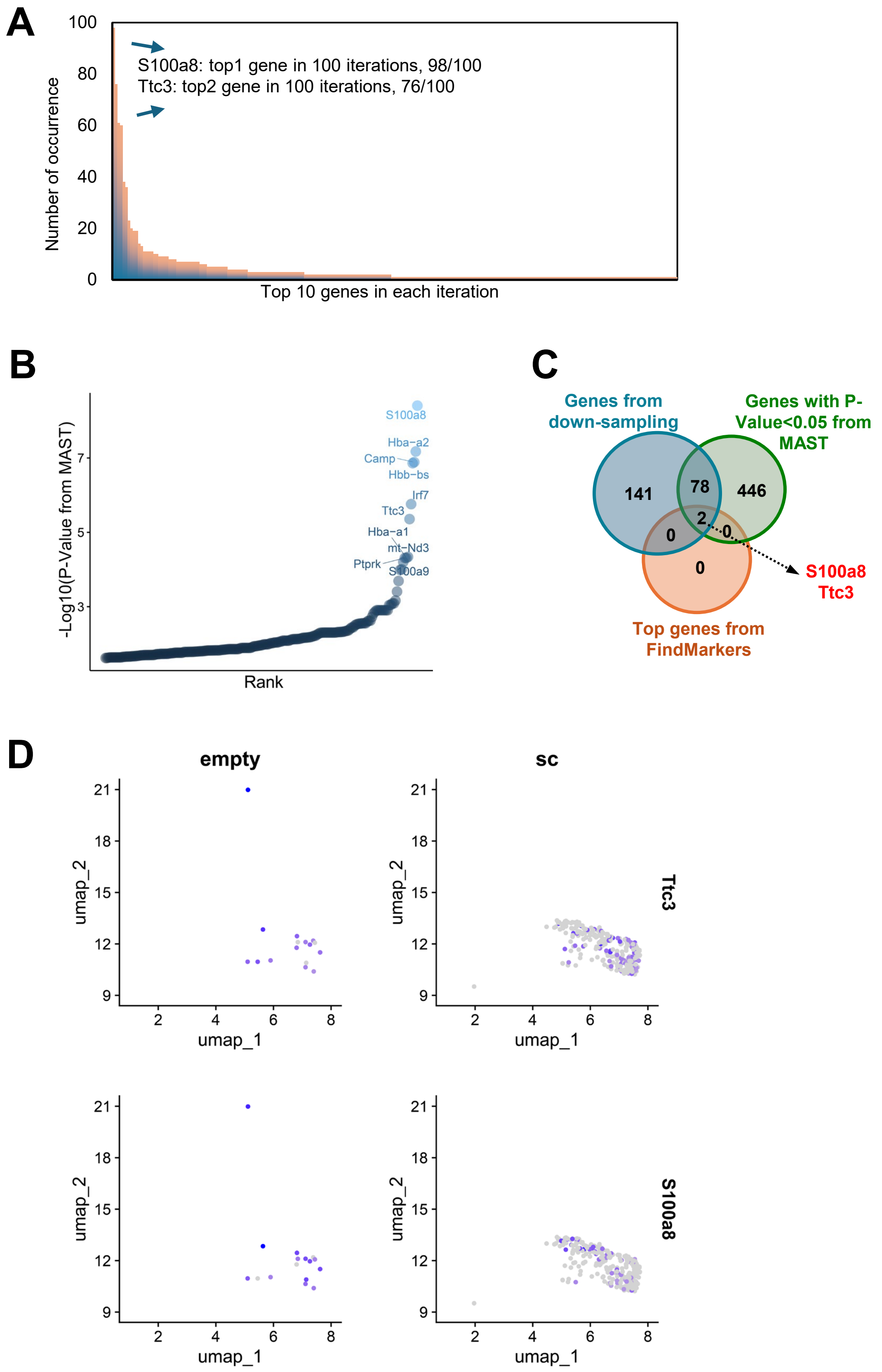

Figure S5. (A) Results on the occurrence of genes in down-sampling analysis. (B) Result from MAST tool for DEG analysis. (C) Overlap of upregulated genes in the Empty group as determined by three different analytical methods. (D)Ttc3 and S100a8 expression in NK cells from Empty and Sc groups.

Figure S6

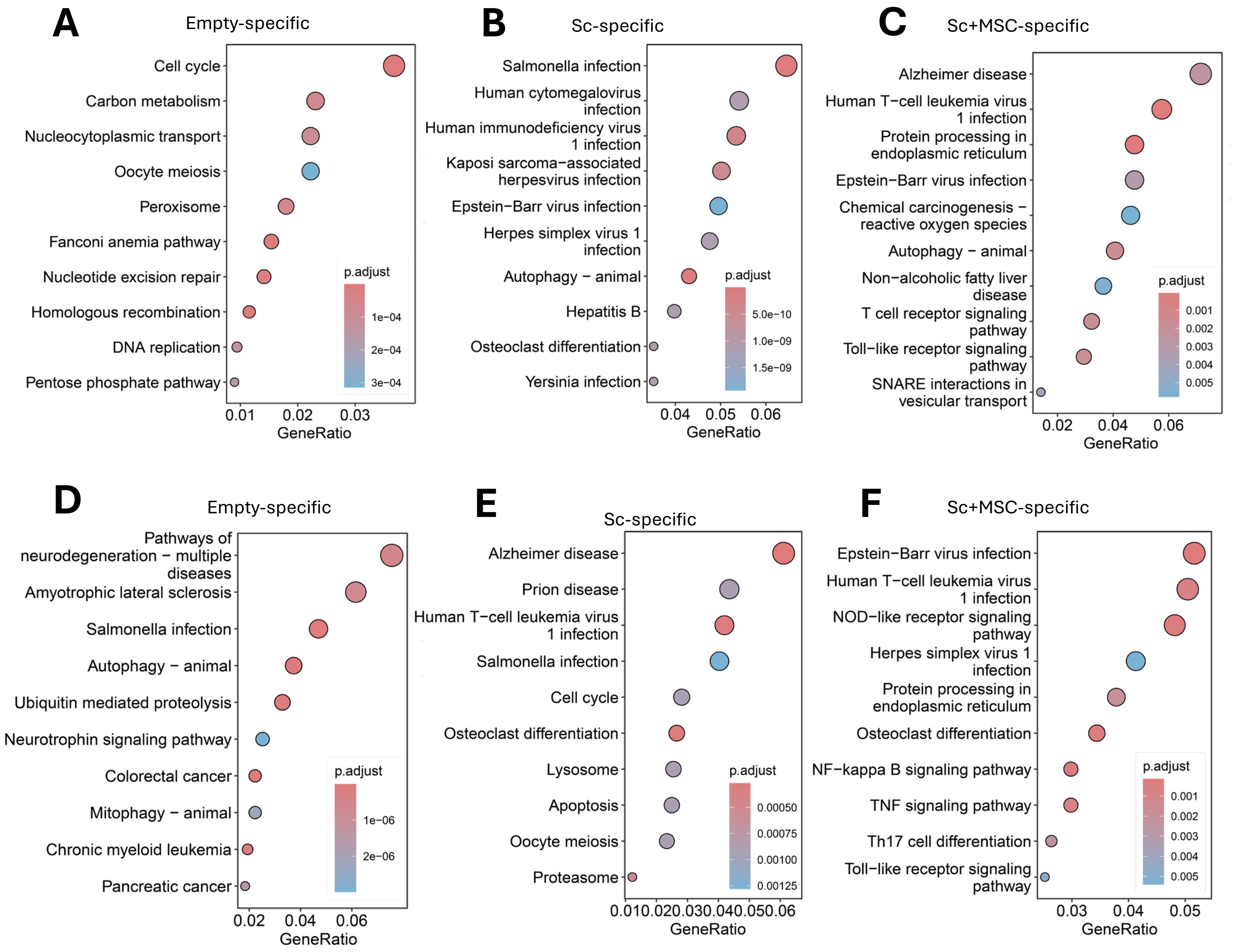

Figure S6. KEGG pathway analysis on the (A) Empty-specific genes, (B) Sc-specific genes, and (C) Sc+MSC-specific genes in DCs. KEGG pathway analysis on the (E) Empty-specific genes, (F) Sc-specific genes, and (G) Sc+MSC-specific genes in NK cells.

Figure S7

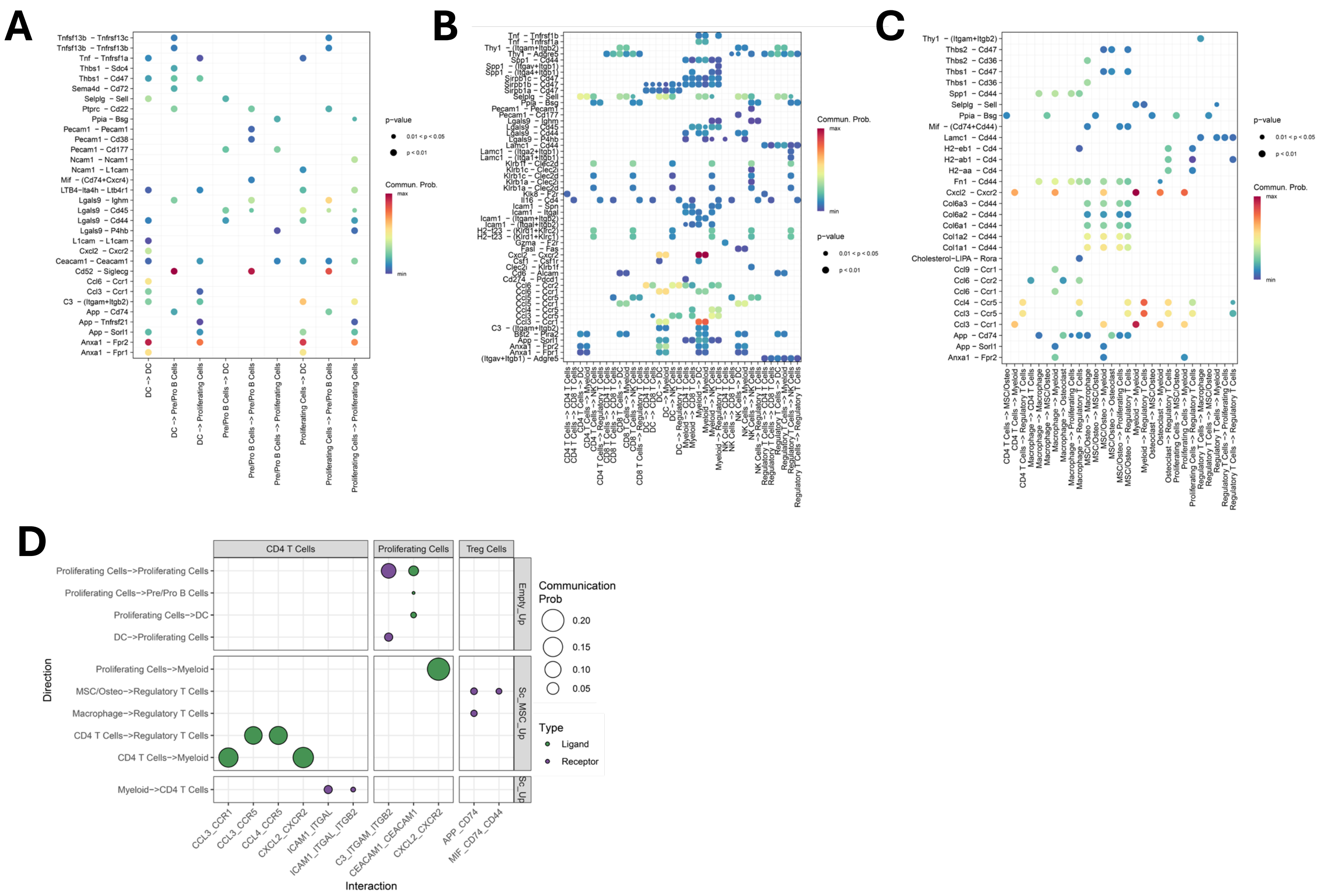

Figure S7. Interaction details in the (A) Empty group, (B) Sc group, and (C) Sc+MSC group. (D) Specific upregulated genes in the three groups contributed to cell-cell communications.
